# Anti-PD-L1/PD-L2 therapeutic vaccination in untreated chronic lymphocytic leukemia patients with unmutated IgHV

**DOI:** 10.3389/fonc.2022.1023015

**Published:** 2022-11-22

**Authors:** Uffe Klausen, Jacob Handlos Grauslund, Nicolai Grønne Dahlager Jørgensen, Shamaila Munir Ahmad, Merete Jonassen, Stine Emilie Weis-Banke, Evelina Martinenaite, Lone Bredo Pedersen, Thomas Landkildehus Lisle, Anne Ortved Gang, Lisbeth Enggaard, Morten Hansen, Morten Orebo Holmström, Özcan Met, Inge Marie Svane, Carsten Utoft Niemann, Lars Møller Pedersen, Mads Hald Andersen

**Affiliations:** ^1^ National Center for Cancer Immune Therapy (CCIT-DK), Department of Oncology, Copenhagen University Hospital Herlev, Herlev, Denmark; ^2^ Department of Hematology, Rigshospitalet, Copenhagen University Hospital, Copenhagen, Denmark; ^3^ Department of Immunology and Microbiology, University of Copenhagen, Copenhagen, Denmark; ^4^ Department of Hematology, Zealand University Hospital, Roskilde, Denmark

**Keywords:** PD-L1, PD-L2, chronic lymphocytic leukemia (CLL), immunotherapy, cancer vaccine

## Abstract

Chronic lymphocytic leukemia (CLL) patients with unmutated immunoglobulin heavy chain (IgHV) are at risk of early disease progression compared to patients with mutated IgHV. As a preventive strategy, we treated 19 previously untreated CLL patients with unmutated IgHV in a phase 1/2 trial (clinicaltrials.gov, NCT03939234) exploring the efficacy and toxicity of a therapeutic cancer vaccine containing peptides derived from programmed death ligand 1 (PD-L1) and ligand 2 (PD-L2), hoping to restore immunological control of the disease. According to the International Workshop on Chronic lymphocytic Leukemia (iwCLL) response criteria, no patients obtained a response; however, during follow-up, one patient had complete normalization of the peripheral lymphocyte count and remained in biochemical remission after a follow-up time of 15 months. At the end of treatment, one patient had progressed, and 17 patients had stable disease. During follow-up with a median time of 23.5 months since inclusion, seven patients had progressed, and eight patients had stable disease. The median time to first treatment (TTFT) from diagnosis was 90.3 months with a median follow-up time of 50.1 months. This apparent favorable outcome in TTFT needs to be investigated in a randomized setting, as our population may have been biased. More than 80% of patients obtained vaccine-specific immune responses, confirming the immunogenicity of the vaccine. The vaccine was generally well tolerated with only grade I–II adverse events. Although there were some signs of clinical effects, the vaccine seems to be insufficient as monotherapy in CLL, possibly due to a high tumor burden. The efficacy of the vaccine should preferably be tested in combination with novel targeted therapies or as a consolidating treatment.

## Introduction

Chronic lymphocytic leukemia (CLL) is a heterogenic disease with varying clinical presentation and prognosis. Several prognostic factors have been identified, and so far, TP53 alterations and immune globulin heavy chain (IgHV) mutation status seem to be the most important (1). Unmutated IgHV is present in approximately 50% of patients and is associated with a shorter time to first treatment, shorter progression-free survival, overall survival, and a higher rate of transformation to aggressive lymphomas (1, 2). Standard treatments are not curative and associated with toxicity, and to date, no effect on survival has been shown in asymptomatic patients with low tumor burden, which is why the standard practice is to keep patients in observation (3). This watch-and-wait paradigm is now being rechallenged, as we are able to identify patients with increased risk of progression and an increasing repertoire of new drugs with more tolerable toxicity (4). Another challenge for CLL patients is their susceptibility to infections due to an impaired systemic immune system (5). This immune dysfunction seems to be orchestrated by CLL cells and is believed to play a role in impaired cancer immune surveillance (6, 7). Several factors are in play, but a key player in especially T-cell dysfunction in CLL is the immune checkpoint programmed death receptor 1 (PD1) and the ligands programmed death ligand 1 (PD-L1) and programmed death ligand 2 (PD-L2) (8, 9). These ligands are part of natural shutdown mechanisms after an inflammatory response but are often expressed by cancer cells, which are thereby able to escape immune surveillance (10). PD-L1 and PD-L2 have been identified on both CLL cells and other immune regulatory cells recruited by the CLL-induced inflammation (7, 11, 12). Overcoming the immune dysfunction in CLL might be crucial to reinstate tumor immune surveillance. Naturally occurring, pro-inflammatory T cells, known as anti-regulatory T cells (anti-Tregs), are able to recognize the self-antigens expressed by regulatory cells and are believed to be involved in the regulation of normal inflammation (13). Such anti-Tregs can also recognize PD-L1 and PD-L2 and can directly react toward both malignant and non-malignant cells expressing these epitopes (14–17). The anti-Tregs can be mobilized by target-specific peptide vaccines, and their anti-cancer effect has been explored in several malignancies (18–21). Noteworthy is the recent results from a phase 1/2 vaccination study combining PD-L1 and indoleamine-2,3-dioxygenase (IDO)-derived peptides in combination with the anti-PD-1 monoclonal antibody nivolumab in patients with malignant melanoma (22). This combination induced high response rates and long progression-free survival when compared to historical controls receiving nivolumab alone (22). In a phase 1 study, we explored the feasibility of a vaccine containing PD-L1- and PD-L2-derived peptides in patients with heavily treated follicular lymphoma. Here, we successfully activated PD-L1-and PD-L2-specific T cells with limited toxicity. Further, we observed some interesting early clinical signals with one patient obtaining a complete response (CR) and additional long-term disease stabilizing effects in several patients, encouraging us to unroll this study in CLL (21). Here, we evaluated the efficacy and safety of the PD-L1 and PD-L2 vaccines in treatment-naïve CLL patients with unmutated IgHV.

## Material and methods

### Study design and patients

This study is a single-center, single-arm, phase 1/2 study investigating the efficacy of a PD-L1- and PD-L2-targeting peptide vaccine in treatment-naïve CLL patients with unmutated IgHV (NCT03939234). The study was performed at Herlev University Hospital and approved by the ethics committee of the capital region (H-19009723); The Danish Medicines Agency (EudraCT: 2018-004869-14) and the Danish Data Protection Agency (VD-2019-231). We conducted the study according to the Declaration of Helsinki, and all patients provided written informed consent. Additionally, the study was externally monitored according to good clinical practice (GCP).

The key inclusion criteria were diagnosis of CLL according to the World Health Organization classification with unmutated IgHV gene assessed according to the European Research Initiative on CLL (ERIC) guidelines (23). Patients could not have received prior CLL-directed treatments, and patients with treatment indications according to the International Workshop on Chronic lymphocytic Leukemia (iwCLL) criteria were excluded. Patients with other malignant diseases requiring treatment or autoimmune diseases were excluded. Full inclusion and exclusion criteria are provided in **Supplementary Table 1**.

The primary endpoint was the clinical response to the vaccine according to guidelines by the iwCLL (3). The secondary endpoint was immune responses toward the vaccine assessed by enzyme-linked immunospot (ELISPOT) and flow cytometry. The tertiary endpoint was a registration of adverse events (AEs), which were systematically scored according to Common Terminology Criteria for Adverse Events (CTCAE) 4.03 (24). Hematological AEs were graded using the iwCLL criteria.

### The vaccine composition and treatment schedule

The vaccine contained 100 µg of a PD-L1-derived peptide (FMTYWHLLNAFTVTVPKDL) and 100 µg of a PD-L2-derived peptide (SLELQLHQIAALFTVTVPKEL) as previously described (21), both acquired from PolyPeptide Laboratories, Strasbourg, France (16, 25). The peptides were dissolved in 500 μl of water with 20% dimethyl sulfoxide (DMSO) and mixed with 500 µl of the adjuvant Montanide ISA-51 (Seppic Inc., Paris, France) immediately before injection, giving a total injection volume of 1 ml. Nine injections were scheduled to be administered over 6 months, with the first six vaccines administered bi-weekly and the final three vaccines given monthly (**Supplementary Figure 1**).

### Clinical response evaluation

According to the iwCLL criteria, the patients were evaluated by a CT scan before and after treatment along with monitoring of hematological parameters. Disease in the bone marrow was not assessed. For nodal tumor burden measurements on CT scans, the largest diameter from representative target lymph nodes from each lymph node region was measured, and the sum of diameters was compared from baseline to end of treatment (EOT). Due to the accessibility of circulating lymphocytes, these were measured before every vaccine during treatment. Time to first treatment was defined as the time from diagnosis until treatment indication by the iwCLL criteria. Patients were censored at CLL-unrelated death, at treatment with steroids, and if lost to follow-up.

### Preparation of blood and isolation of B cells

Blood samples for the trial-specific analysis described below were taken at baseline as well as after three, six, and nine vaccinations. Peripheral blood mononuclear cells (PBMCs) were extracted from full blood using lymphoprep (STEMCELL Technologies, Vancouver, BC, Canada) in leucosep tubes (Greiner Bio-One, Kremsmünster, Austria) and frozen in 90% human serum and 10% DMSO using controlled rate CoolCell boxes (Brooks Life Sciences, Chelmsford, MA, USA). The PBMCs were stored at −140°C until they were thawed for analysis.

### Interferon-gamma enzyme-linked immunospot

To evaluate vaccine-induced immune responses, we measured the interferon-gamma (IFNγ) release from T cells stimulated with either the PD-L1 or PD-L2-derived vaccine peptides as we have previously described (14, 16). In short, the assay was performed on PBMCs thawed and stimulated once *in vitro* with target peptide to increase assay sensitivity (26). This was performed in 24-well plates with 0.5 ml of X-VIVO medium and left at room temperature for 2 h. Then, we added 1.5 ml of X-VIVO medium with 5% human serum and incubated the plates at 37°C. IL-2 was added the following day, reaching a concentration of 120 U/ml. The plates were incubated for additional 13 days. Then, 2.5–3.5 × 10^5^ cells were then added per well for triplicate analyses in 96-well polyvinylidene difluoride (PVDF) plates (MultiScreen, MAIP N45; Merck Millipore, Burlington, MA, USA) pre-coated with anti-IFNγ-mAb (mAb 1-DIK, Mabtech, Stockholm, Sweden). The respective peptides were added to the wells, and the plates were incubated at 37°C for approximately 24 h. The plates were then washed thoroughly, and the secondary anti-IFNγ-mAb was added and incubated for 2 h at 37°C. The plates were then washed, and streptavidin-enzyme conjugate (AP-Avidin; Calbiochem/Invitrogen Life Technologies, Carlsbad, CA, USA) was added to the wells followed by incubation for 1 h. Then, we added the enzyme substrate NBT/BCIP (Invitrogen Life Technologies) to induce the IFNγ spots. These were counted with the ImmunoSpot series 2.0 Analyzer (Cellular Technology Limited, Cleveland, OH, USA). Wells with spots too numerous to count were set to 500 spots.

### Flow cytometry

For flow cytometric analyses, we used a panel encompassing T-cell subsets such as naïve T cells, memory T cells, regulatory T cells, and senescent T cells. We used another panel to characterize circulating myeloid cells including NK cells, antigen-presenting cells, and monocytic myeloid-derived suppressor cells (mMDSCs) along with CLL B cells and PD-L1- and PD-L2-positive cells. A full list of antibodies is provided in **Supplementary Table 2**, and the gating strategy is illustrated in **Supplementary Figures 2**, **3**. NovoCyte Quanteon Flow Cytometer (Agilent, Santa Clara, CA, USA) was used for cell acquisition, and FlowJo v 10.7.1 was used for gating.

### Statistics

Clinical response was evaluated based on the iwCLL response criteria (3). ELISPOT responses were calculated using the non-parametric distribution-free resampling (DFR) and DFR (2×) methods as described by Moodie et al. (27) For ELISPOT results in duplicates and wells too numerous to count, we used an empirical response definition of two times above background. DFR calculations were performed in R v. 3.6.1. The Wilcoxon test was used to compare ELISPOT responses from baseline with the other timepoints. We used the one-way ANOVA test to test the significance of changes in immune populations over time identified by flow cytometry, with only significant results reported. Graphs and statistical calculations were performed in GraphPad Prism 9.0.0.

## Results

### Patients

Due to a stop in recruitment with the arrival of the COVID-19 pandemic, 19 out of the intended 20 patients were included from May 2019 to February 2020. The median time from diagnosis to inclusion was 26 months (range, 39 days–11.6 years). Patient characteristics are listed in **Table 1**. Most patients had intermediate-risk disease according to the CLL international prognostic index (1). The median lymphocyte count at inclusion was 24 × 10^9^ cells/L, ranging from 4.4 to 170 × 10^9^ cells/L. Seventeen patients completed the full vaccination schedule of nine vaccines, as one patient was diagnosed with lung cancer needing treatment and another patient had disease progression during the vaccination schedule. PBMCs isolated from the patients showed that all patients had increased mRNA expression of PD-L1 and PD-L2 compared to B cells isolated from healthy donors (**Supplementary Figure 4A**). Flow cytometric analysis showed few CLL cells with presumably low surface expression of PD-L1 (0.5% positive B cells) and PD-L2 (2.5% positive B cells) (**Supplementary Figure 4B**).

**Table 1:** Baseline characteristics.

Gender (M:F)	12:7
Median age (range)	70 (53–81)
Performance status	
0	17
1	2
Binet stage	
A	17
B	1
C	1
Cytogenetics	
del(11q)	3
del(13q)	9
del(17p)	0
TP53 mutated	0
Trisomy 12	2
CLL-IPI	
Low	0
Intermediate	17
High	2
Median lymphocyte count (range)	24 × 10^9^ (4.4–170)
Median lymph node tumor burden. Sum of longest diameter in mm (range)	72.5 mm (0–216)
Median time in months since diagnosis (range)	26.1 months (1.3–139.3)

CLL-IPI, International Prognostic Index for Chronic Lymphocytic Leukemia.

### Clinical response

**Figure 1:**
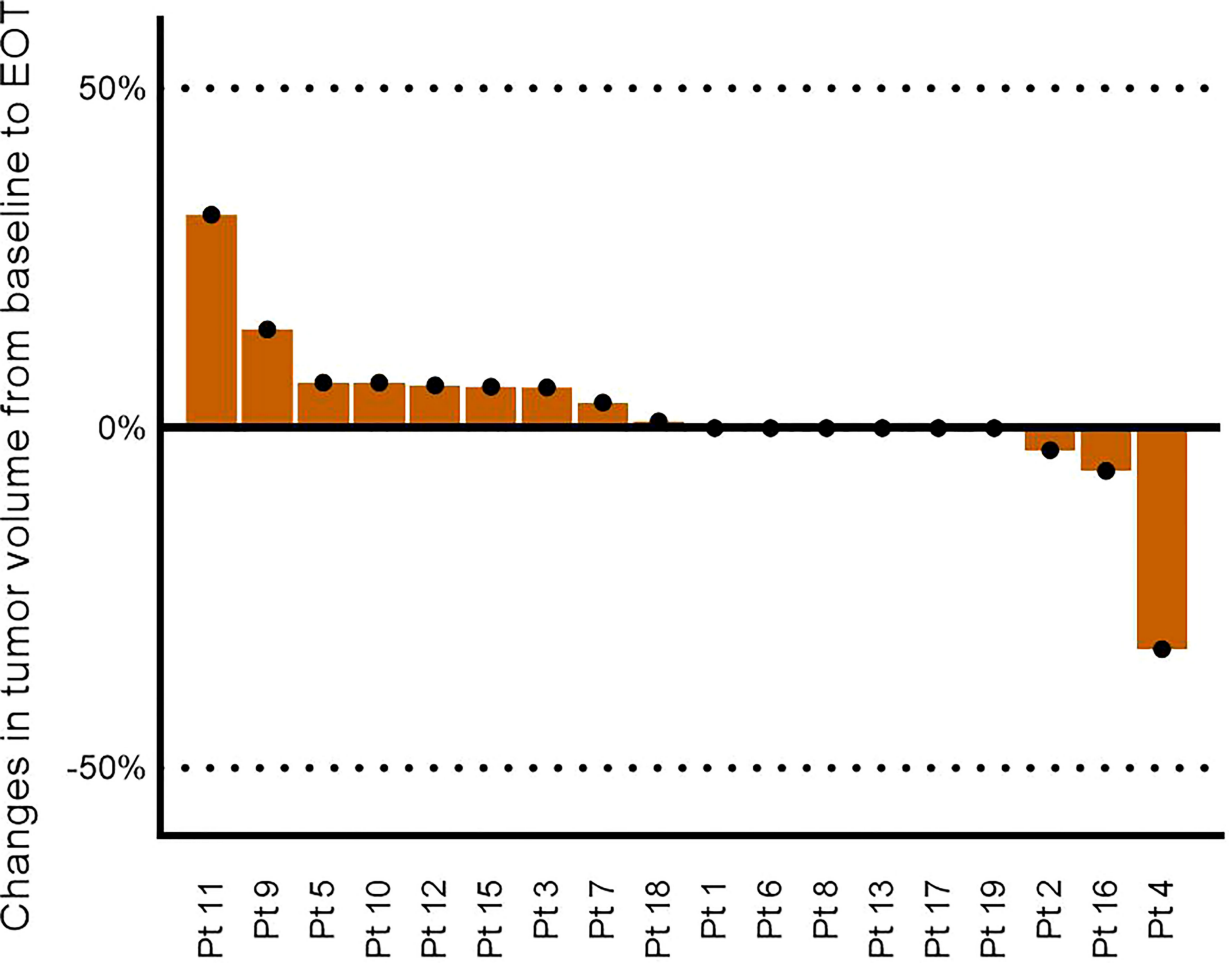
Changes in nodal tumor burden from baseline to end of treatment based on the sum of the largest diameter of target lymph nodes. Pt14 is not included due to exclusion from the study.

**Figure 2:**
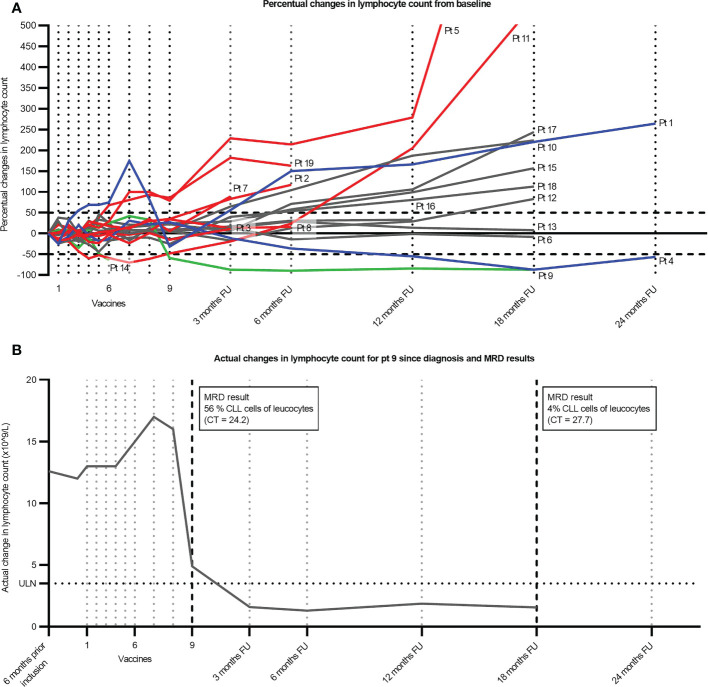
Lymphocyte count for individual patients over time. **(A)** Percentual changes in lymphocyte count from baseline. Red indicates patients who have started the next treatment, and the line indicates patients censored at the start of treatment. Blue indicates that the patient has been treated with steroids for periods during the depicted timespan. Green indicates patients achieving a 50% drop in lymphocyte count without subsequent progression or steroid treatment. The remaining patients are depicted in gray. **(B)** Actual changes in lymphocyte count for Pt9 since diagnosis. A percentual estimate of CLL cells of leucocytes in peripheral blood is indicated by the arrows based on qPCR results from the MRD assay. The fraction of CLL cells is calculated on basis of CT values from samples with different known CLL concentrations as described in the Supplementary Methods. FU, follow-up; ULN, upper limit of normal; CLL, chronic lymphocytic leukemia; MRD, minimal residual disease.

**Figure 3:**
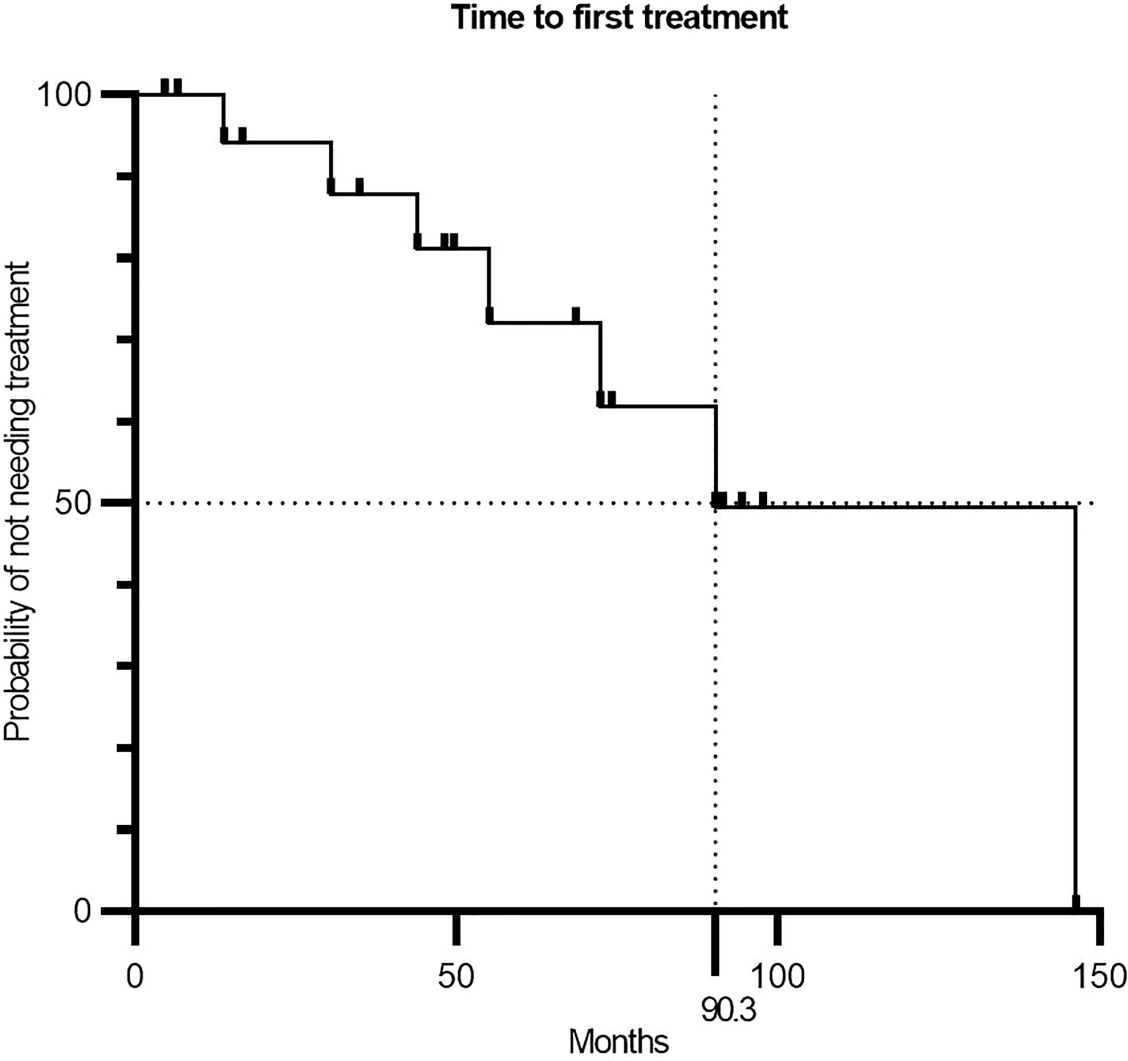
Kaplan–Meier curve showing the time between CLL diagnosis and initiation of first treatment (n = 19). CLL, chronic lymphocytic leukemia.

Taking lymphocyte count, hematological parameters, and nodal tumor burden into account, we did not see any responses qualifying for the criteria defined by iwCLL. One patient had progressive disease (PD), and 17 had stable disease (SD) at EOT, with the last patient excluded due to lung cancer. During the treatment period, we saw a 50% drop in lymphocytes in three patients, but they did not have a concurrent reduction in nodal tumor burden on EOT CT scans, disqualifying them for partial response (**Figures 1**, **2A**). Interestingly, one of these patients (pt9) had a continued lymphocyte reduction, reaching normal levels of peripheral lymphocytes months after EOT. After 18 months of follow-up, the patient was still in biochemical remission (**Figure 2B**). At this stage in the trial, we were unable to further assess the nodal tumor burden with CT scans; however, we evaluated the minimal residual disease (MRD) with RT-qPCR targeting the clonal B-cell receptor using a validated assay by the EuroMRD consortium (Supplementary Methods). MRD was still detectable at 18 months of follow-up and, as expected, had lower copy numbers when compared to EOT marked by the timepoint nine in **Figure 2B**. After a median follow-up time of 23.5 months since inclusion and a median time of 50.1 months since diagnosis, seven patients had PD, and eight patients had SD. Of the remaining four patients, two had received larger doses of steroids, and one was lost to follow-up with SD at the latest visit. We found a median time to first treatment (TTFT) of 90.3 months since the diagnosis (**Figure 3**).

### Immune responses and immune composition

**Figure 4:**
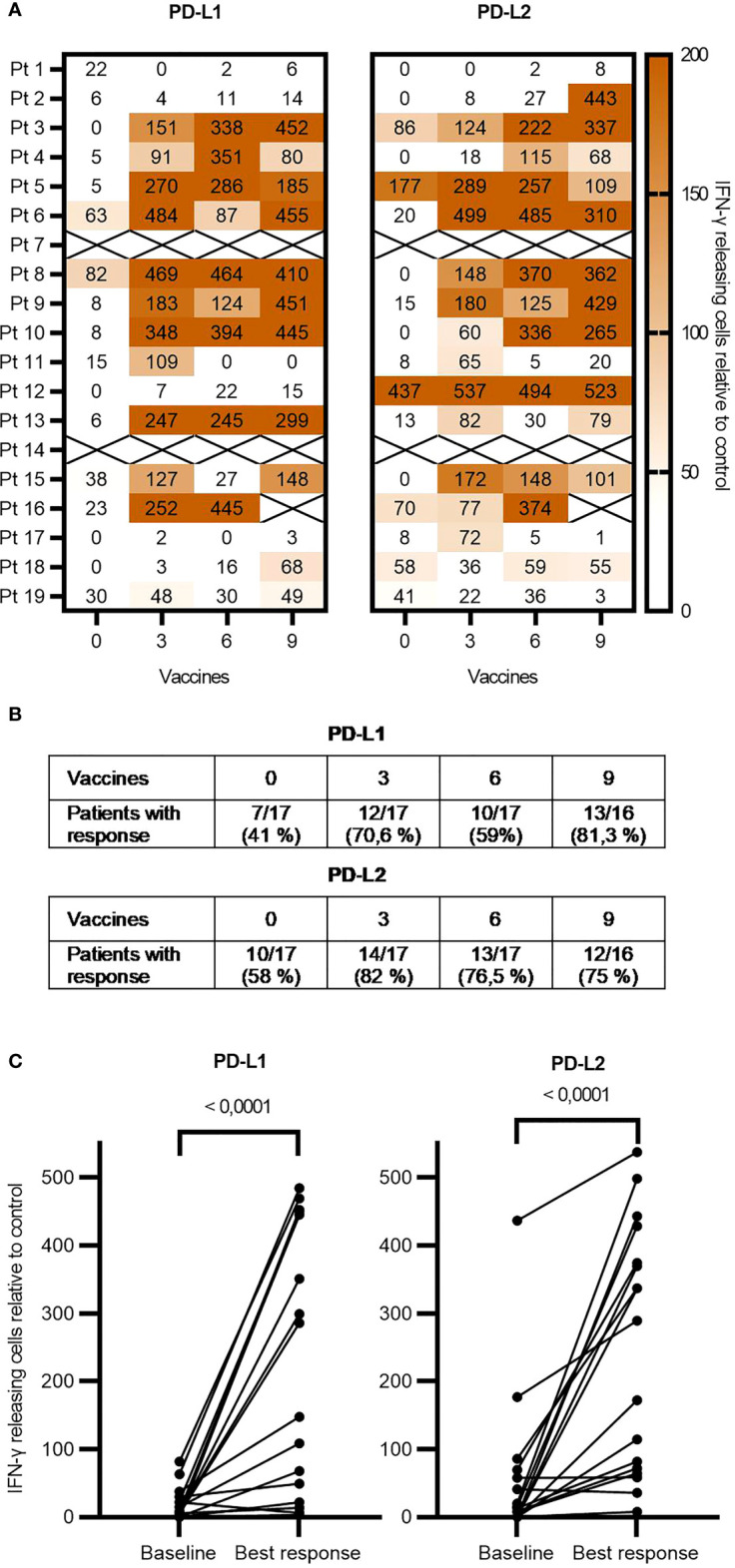
ELISPOT responses of individual patients over time. **(A)** ELISPOT analysis showing the immune responses of each patient at each timepoint during vaccination. The spot count is in relation to the control and based on the mean of triplicates. **(B)** Table showing the fraction of responses at each timepoint counting statistically significant responses defined by DFR, DFR (2×), and the empirical response definition. **(C)** Graph comparing the baseline level of T-cell responses to the best response during vaccination using the Wilcoxon test. ELISPOT, enzyme-linked immunospot; DFR, distribution-free resampling.

**Figure 5:**
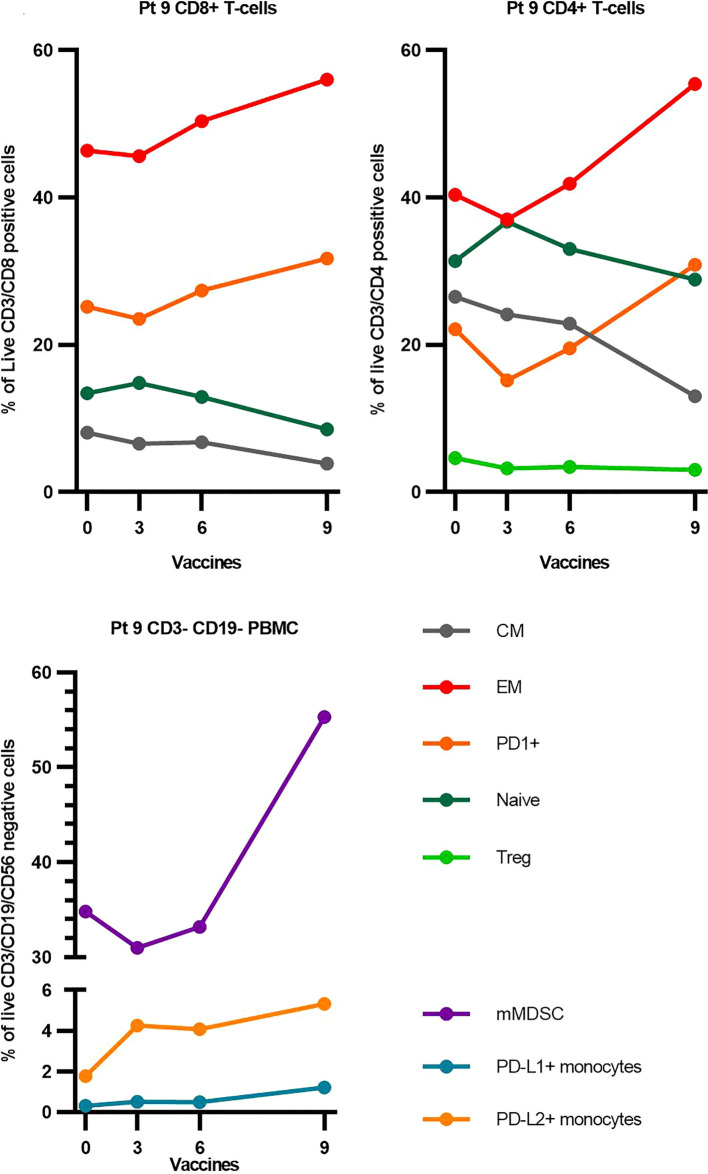
Changes in selected immune subsets for Pt9. For a detailed gating strategy, see **Supplementary Figure 3**. EM, effector memory; CM, central memory; Treg, regulatory T cells; PBMCs, peripheral blood mononuclear cells; mMDSC, monocytic Myeloid Derived Suppressor Cells.

Out of the 19 patients, 17 had their T-cell response evaluated by ELISPOT. In two patients, we failed to culture T cells. Generally, we detected low recognition and weak responses to both peptides at baseline (**Figure 4A**). After the introduction of the vaccine, we saw a significant increase in the number and amplitude of responses (**Figures 4B, C**). The number of statistically significant responses is specified in **Figure 4B**. Pt9, who went into biochemical remission, had responses similar to those of non-responding patients. By flow cytometry, we investigated the impact that the vaccine might have had on common immune subsets. We observed wide interindividual variations in the population sizes of the different immune subsets, but overall, no changes were observed over time within each subset. PD-L1 and PD-L2 are often expressed on monocytic immune cells such as mMDSCs, but we did not observe any changes in the frequency of PD-L1 and PD-L2 expressing monocytes (**Supplementary Figures 5** and **6**). Further examinations of the changes within the subsets of Pt9 showed a shift from central memory and naïve phenotypes toward the activated effector memory phenotype with an increased PD1 expression for both CD4+ and CD8+ T cells (**Figures 5A, B**). Conversely, we observed increased numbers of mMDSCs and PD-L1- and PD-L2 expressing monocytes (**Figure 5C**).

### Adverse events

The vaccine was generally well tolerated, and only grade one and grade two adverse events were directly related to the vaccine by the assessment of the investigators (**Table 2**). The most common adverse reaction recorded was injection site reactions experienced by 79% of patients. Indurations at the injection site were common and remained during follow-up in some patients. These granulomas are known complications of the use of Montanide and have been reported in earlier vaccine trials with this adjuvant (19, 21, 22, 28). Two patients experienced chills in close relation to the vaccine, also a known reaction to Montanide (28). During the study including follow-up, six serious adverse events occurred. Four of these were deemed not related to the vaccine as assessed by the investigators, including one case of severe COVID-19 infection, one pneumonia requiring admission, and a case of ileus requiring surgery. One patient was admitted with autoimmune anemia during progression, and this condition was deemed related to the CLL diagnosis as a common complication (29). In two cases, we could not rule out the relation to the vaccine and rated the events as possibly related, which is not included in **Table 2**. This included a patient who was admitted during investigations for facial nerve palsy. No underlying causes were identified, and symptoms gradually disappeared, confirming idiopathic facial nerve palsy. Another patient experienced debilitating joint and muscle pain toward the end of the vaccination schedule. This patient underwent extensive investigations at the Department of Rheumatology, and despite ongoing steroid-refractory pain, no results were abnormal.

**Table 2:** Adverse events related and possibly related to the vaccine.

	Number of events	Grade	Number of patients, n = 19
Injection site reaction	28	1–2	15 (79%)
Chills	6	1	2 (11%)
Skin infection	1	2	1 (5%)
Night sweats	1	1	1 (5%)
Dizziness	1	1	1 (5%)
Hyperalgesia	2	1	1 (5%)
Insomnia	1	2	1 (5%)
Bursitis	1	2	1 (5%)
Headache	2	1	1 (5%)
Adenitis	1	1	1 (5%)
Frequent urination	1	1	1 (5%)

## Discussion

In this phase 1/2 study exploring the clinical efficacy of a therapeutic cancer vaccine with peptides derived from PD-L1 and PD-L2 in untreated CLL patients with unmutated IgHV, we did not see any responses according to the iwCLL response criteria (3). Three patients had a decline in peripheral lymphocyte counts; however, this change was not accompanied by any changes in the size of lymph nodes. Interestingly, one patient had a normalization of the lymphocyte count a few months after the last vaccine despite an increase in lymph node size on the EOT CT scan a few months earlier. This biochemical remission lasted for at least 15 months, as it was maintained at their last visit. This response is similar to the responses we saw in the previous study in follicular lymphoma, where two patients went into spontaneous remission during follow-up after an initial increase in lymph nodes (21). Similarly, Pt9 had a notable increase in lymph node volume as illustrated in **Figure 1**. Unfortunately, we were not able to investigate the matter further, as lymph node biopsies and further CT scans were not part of the study. It should be noted that Pt9 could have had a spontaneous regression without relation to the vaccine, which is a rare albeit well-described event in CLL (30). The underlying mechanisms in these regressions are believed to be of immunological nature including T-cell reactivity toward the CLL clone. Due to the onset of the regression in Pt9 at the end of the treatment, it is very likely that the PD-L1- and PD-L2-specific T-cell responses induced by the vaccines have reinstated the tumor immune surveillance in this patient. With only one measurable response from 19 patients, we were unable to identify predictive factors for the response. For future records, Pt9 was further characterized by having Binet stage A, del(11q), and del(13q) and being male. Further, Pt9 had relatively low lymphocyte count among participants, and we speculate if the enormous tumor burden often involves billions of CLL cells in the blood, lymph nodes, and bone marrow, and often, extra lymphatic organs can prove too great an obstacle.

We found a median TTFT of 90 months, which is considerably longer than the previously reported median TTFT of 22–52 months for intermediate-risk patients (1). Interestingly, in two recent studies, Baumann et al. and Morabito et al. have documented the relationship between lymphocyte doubling time and TTFT in CLL (31, 32). However, the current study population was biased, as some patients were included long after the diagnosis, indicating indolent disease when compared to patients who may already have received treatment, making any conclusion on this matter speculative. Thus, the favorable outcome in TTFT found here should be investigated in a randomized setting along with further investigations including lymphocyte doubling times into whether a subgroup of patients is more likely to benefit from this type of treatment. The patients with a 50% decrease in lymphocyte count not treated with corticosteroids had an initial lymphocyte count under the median of 24 × 10^9^/L, indicating that a small circulating tumor burden might be one factor of importance. This supports the notion of the inability of therapeutic cancer vaccines to induce a clinical response in patients with a high tumor burden. We have previously planned our vaccination trials in such a way that the vaccine has been either preceded by cytoreductive therapy or administered alongside other active substances (19, 21). Further, we have focused on indolent and chronic malignancies such as follicular lymphoma, multiple myeloma, and chronic myeloid neoplasms, allowing time for the vaccine to establish proper immune responses and tip the immunological balance toward tumor elimination (19–21). Here, we introduced the vaccine in untreated patients as monotherapy, a valuable opportunity to obtain data on the vaccine alone, but we realize that the immunological effect of therapeutic cancer vaccines as monotherapy might be too weak in order to overcome the tumor burden in this systemic cancer disease. Hence, future strategies should involve either prior conditioning therapies to reduce the tumor burden as we did in follicular lymphoma (21) or adjuvant therapy with effective or even synergistic agents such as the IDO- and PD-L1-derived peptide vaccines in combination with nivolumab in malignant melanoma. We also speculate if the shift in T-cell subsets detected in Pt9 is a prerequisite for a clinical response, as the extent of these changes was unique for this patient and similar to what we detected in follicular lymphoma patients believed to respond to the vaccine in our previous trial (21). These changes in the immune compartment could be a prerequisite for clinical response and should be investigated further. In this regard, we did see a large faction of patients establishing immune responses toward the target peptides but without a concurrent clinical response according to the accepted response criteria. This could also suggest that the targets of the vaccine have too little significance in the overall pathophysiology of CLL. This hypothesis is supported by the poor effect of PD-1 blockade in CLL, where mainly patients with Richter’s transformation seem to benefit from this treatment (33, 34). We did detect expression of PD-L1 and PD-L2 in CLL cells by RT-qPCR, but very low to no expression was detected by flow cytometry. The effect of the vaccine to the immune modulation may therefore mainly be through the elimination of PD-L1 and PD-L2 expressing bystander cells, which we believe is an equally crucial mechanism of action of the vaccine. A study in diffuse large B-cell lymphoma (DLBCL) have shown an association between a high mutation rate of IgHV and the amplification of PD-L1/PD-L2 (9p24.1), which could explain our limited detection of the ligands in this study; however, this has not been investigated in CLL (9, 35). Another study has found that the PD-L1 expression of CLL cells is increased in the proliferative zones in lymph nodes (9). As we only evaluate circulating CLL cells, we might miss the dimension of the immunological effects of the vaccine happening in the lymph nodes. It is increasingly acknowledged that the tumor microenvironment should be addressed when treating cancer. With CLL having both a leukemic and a solid compartment, the disease has a vast and variable tumor environment for the vaccine to overcome. We know that current therapies have a different impact in each compartment, where residual disease in lymph nodes poses a challenge to chemoimmunotherapy, and ibrutinib has been shown to mobilize CLL cells from lymph nodes to the circulation through the regulation of the chemokine receptor CXCR4 (36, 37). These results encourage future vaccine trials to secure lymph node/tumor biopsies to elucidate the effects of the vaccine on different microenvironmental niches. Another challenge to consider regarding T-cell-dependent immunotherapy in CLL is the characteristic exhausted T-cell profile (7, 38). Treatment-naïve CLL patients have inferior responses when compared to healthy individuals to a variety of vaccines including influenza, pneumococcal, and COVID-19 vaccines (39–41). Chimeric antigen receptor T-cell therapy, another treatment relying on T-cell fitness, has shown much lower response rates in CLL when compared to response rates of acute lymphoblastic leukemia patients (42, 43). The role of T-cell dysfunction has been underscored by murine CLL models, where pre-conditioning of mice with ibrutinib, which has T-cell restoring properties, improved the T-cell product resulting in improved tumor response (44). Being aware of this, we suspected that the poor condition of T cells might be an issue for the vaccine to mount an adequate T-cell immune response. Nonetheless, the vaccines induced T-cell responses similar in frequency and amplitude to responses identified in previous cancer vaccine trials from our institution (19–22). Hence, overcoming the vast tumor burden might be a greater issue in CLL. Comprehending the abovementioned challenges, we believe that the future strategy in CLL should primarily address the vast tumor burden and the immune dysfunction when applying T cell-dependent immunotherapy, and synergetic combinations should be pursued. As ibrutinib has immune restorative effects, reduces tumor burden, and induces alterations in the tumor microenvironment, it seems apparent to explore a combination of ibrutinib or other tyrosine kinase inhibitors with this vaccine in CLL. Another option could be to use the vaccine as consolidation therapy for MRD-positive patients or as maintenance therapy to maintain remission as we explored in the phase 1 trial.

## Conclusion

In this trial, we did not see any immediate clinical responses to the PD-L1/PD-L2 peptide vaccine according to the iwCLL response criteria. However, one patient achieved a durable biochemical remission, indicating a possible long-term disease stabilizing in this patient. With patients still in treatment-free observation, we continue to monitor for long-term disease-stabilizing effects. The vaccine is tolerable and induces immune responses toward vaccination epitopes in most patients. Based on the results described above, we conclude that future immunotherapeutic approaches in CLL should take the high tumor burden into account along with the dysfunctional T-cell compartment in order to increase the chance of clinical response to this form of therapy.

## Data availability statement

The raw data supporting the conclusions of this article will be made available by the authors, without undue reservation.

## Ethics statement

The studies involving human participants were reviewed and approved by the ethics committee of the capital region (H-19009723). The patients/participants provided their written informed consent to participate in this study.

## Author contributions

All authors contributed to the manuscript in regard to either conceptualizing and designing the trial or collecting, analyzing, and interpreting data. All authors contributed to the article and approved the submitted version.

## Funding

The work was funded by grants from the Independent Research Fund Denmark (0134-00072B), the Danish Cancer Society (F204-A12583), and Copenhagen University (PhD stipends) and through a research agreement between IO Biotech ApS and the National Center for Cancer Immune Therapy (CCIT-DK), Herlev Hospital, Capital Region, Denmark.

## Acknowledgments

We would like to express our gratitude to the CLL patients participating in this trial.

## Conflict of interest

The first author UK was employed at National Center for Cancer Immune Therapy CCIT-DK, supported by a research agreement with IO biotech APS during the trial. Further, the study is part of a PhD thesis by UK, who answers to MA, his primary supervisor and employer. MA is named as an inventor on various patent applications relating to therapeutic uses of PD-L1 and PD-L2 peptides. These patent applications are assigned to the company IO Biotech, which is developing immune-modulating cancer treatments. MA and IS have cofounded IO Biotech and are shareholders and active advisors in the company. CN received research funding and/or consultancy fees outside this study from AbbVie, AstraZeneca, Janssen, CSL Behring, BeiGene, Genmab, Takeda, and Octapharma. Large parts of the study have taken place at CCIT-DK, which is an organization that has an economical interest in the tested vaccines according to the patent laws in Denmark. JG, SW-B, SA, MH, MOH, and ÖM were employed at CCIT-DK during the trial, answering to either IMS or MHA. EM is an employee at IO Biotech.

The remaining authors declare that the research was conducted in the absence of any commercial or financial relationships that could be construed as a potential conflict of interest.

## Publisher’s note

All claims expressed in this article are solely those of the authors and do not necessarily represent those of their affiliated organizations, or those of the publisher, the editors and the reviewers. Any product that may be evaluated in this article, or claim that may be made by its manufacturer, is not guaranteed or endorsed by the publisher.
