# Supplementary material for: Anti-PD-L1/PD-L2 therapeutic vaccination in untreated chronic lymphocytic leukemia patients with unmutated IgHV
Source: Front Oncol. 2022 Nov 22;12:1023015. doi: 10.3389/fonc.2022.1023015 (PMC9723164; doi:10.3389/fonc.2022.1023015)

Supplementary Materials

Supplementary Table 1: Inclusion and Exclusion Criteria

Inclusion Criteria	Exclusion Criteria
CLL according to national guidelines	Other active malignant diseases requiring treatment.
Unmutated IGHV gene according to ERIC recommendations	Significant medical condition per investigators judgement e.g., severe Astma/COLD, poorly regulated heart condition, insulin dependent diabetes mellitus.
No prior CLL directed treatment	Acute or chronic viral/bacterial infection e.g., HIV, CMV, EBV, hepatitis or tuberculosis
Age $\geq$ 18	Serious known allergies or earlier anaphylactic reactions.
ECOG performance status of 0 or 1	Known sensibility towards Montanide ISA 51
No life-threatening conditions	Any active autoimmune diseases e.g., autoimmune neutropenia, thrombocytopenia or hemolytic anemia, systemic lupus erythematosus, scleroderma, myasthenia gravis, autoimmune glomerulonephritis, autoimmune adrenal deficiency, autoimmune thyroiditis etc.
Bone marrow function: Neutrophilocytes > 1,0 x 10^9/L; Platelets > 100 x 10^9/L	Pregnant and breastfeeding women.
Renal function: Creatinine < 300 µmol/l	Psychiatric disorders that according to the investigator could influence compliance.
Liver function: Aspartate Aminotransferase < 100 U/L	Treatment with other experimental drugs
For fertile women: agreement to use contraceptive methods with a failure rate of < 1% per year during the treatment period and for at least 120 days after the last treatment.	
For men: agreement to use contraceptive measures and agreement to refrain from donating sperm.	

Supplementary Table 2

T cell panel			
**Antigen**	**Fluorochrome**	**Clone**	**Manufacturer**
**PD-1**	**PE-Cy7**	**EH12.1**	**BD Bioscience, Franklin lakes, New Jersey, USA**
**TCR γδ**	**BV421**	**B1**	**BD Bioscience**
**CD3**	**BV786**	**SK7**	**BD Bioscience**
**CD4**	**BV510**	**SK3**	**BD Bioscience**
**CD8**	**PE-CF594**	**HIT8α**	**BD Bioscience**
**CD25**	**APC-R700**	**2A3**	**BD Bioscience**
**CD27**	**BV711**	**L128**	**BD Bioscience**
**CD28**	**BV605**	**L293**	**BD Bioscience**
**CD45RA**	**APC**	**HI100**	**BD Bioscience**
**CD57**	**FITC**	**NK-1**	**BD Bioscience**
**CD127**	**BV650**	**A019D5**	**Nordic Biosite AB, Täby, Sweden**
**CCR7**	**PE**	**G043H7**	**Nordic Biosite**
**NIR (Live/dead)**	**APC-Cy7**		**Thermo Fischer Scientific, Waltham, Massachusetts, USA**
**PBMC panel**			
**CD1c**	**APC**	**L161**	**Nordic Biosite**
**CD3**	**BV786**	**SP34-2**	**BD Bioscience**
**CD11c**	**BV650**	**B-ly6**	**BD Bioscience**
**CD14**	**PE-Daz594**	**HCD14**	**Nordic Biosite**
**CD16**	**FITC**	**3G8**	**Nordic Biosite**
**CD19**	**PE**	**4G7**	**Nordic Biosite**
**CD33**	**BV510**	**WM53**	**BD Bioscience**

CD56	PE-Cy7	NCAM16.2	BD Bioscience
CD123	BV605	7G3	BD Bioscience
HLA-DR	PerCP-Cy5.5	G46-6	BD Bioscience
PD-L1	BV711	29E.2A3	Nordic Biosite
PD-L2	BV421	MIH18	BD Bioscience
CD5	APC-R700	UCHT2	BD Bioscience
NIR (Live/dead)	APC-Cy7		Thermo Fischer Scientific

Supplementary Table 3: List of Primers

GENE	PROTEIN	PRIMER ID	SOURCE
CD274	PD-L1	Hs001125296_m1	Thermo Scientific
PDCD1LG2	PD-L2	Hs00228839_m1	Thermo Scientific
POLR2A	POLR2A	Hs00172187_m1	Thermo Scientific

Supplementary Figure 1

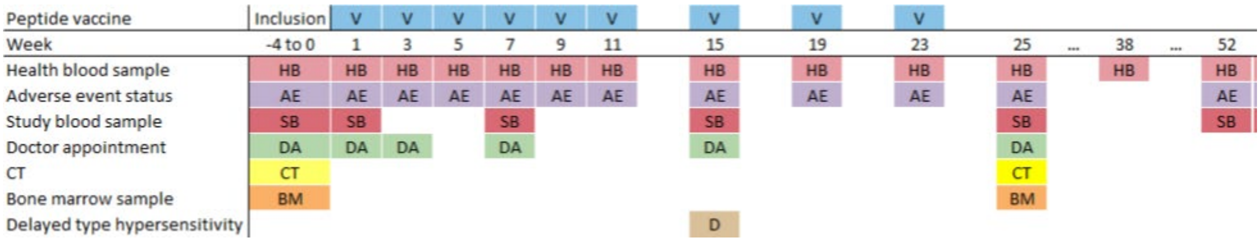

Supplementary Figure 2: Gating Strategy

A

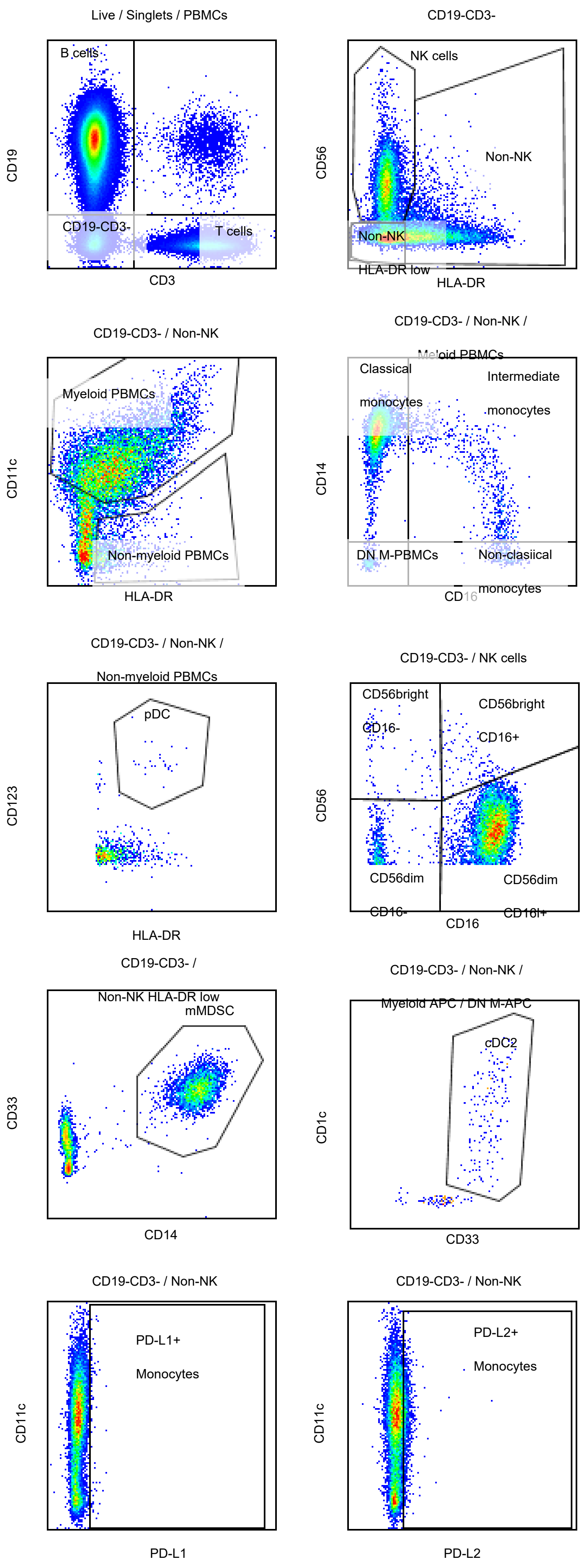

Supplementary Figure 3

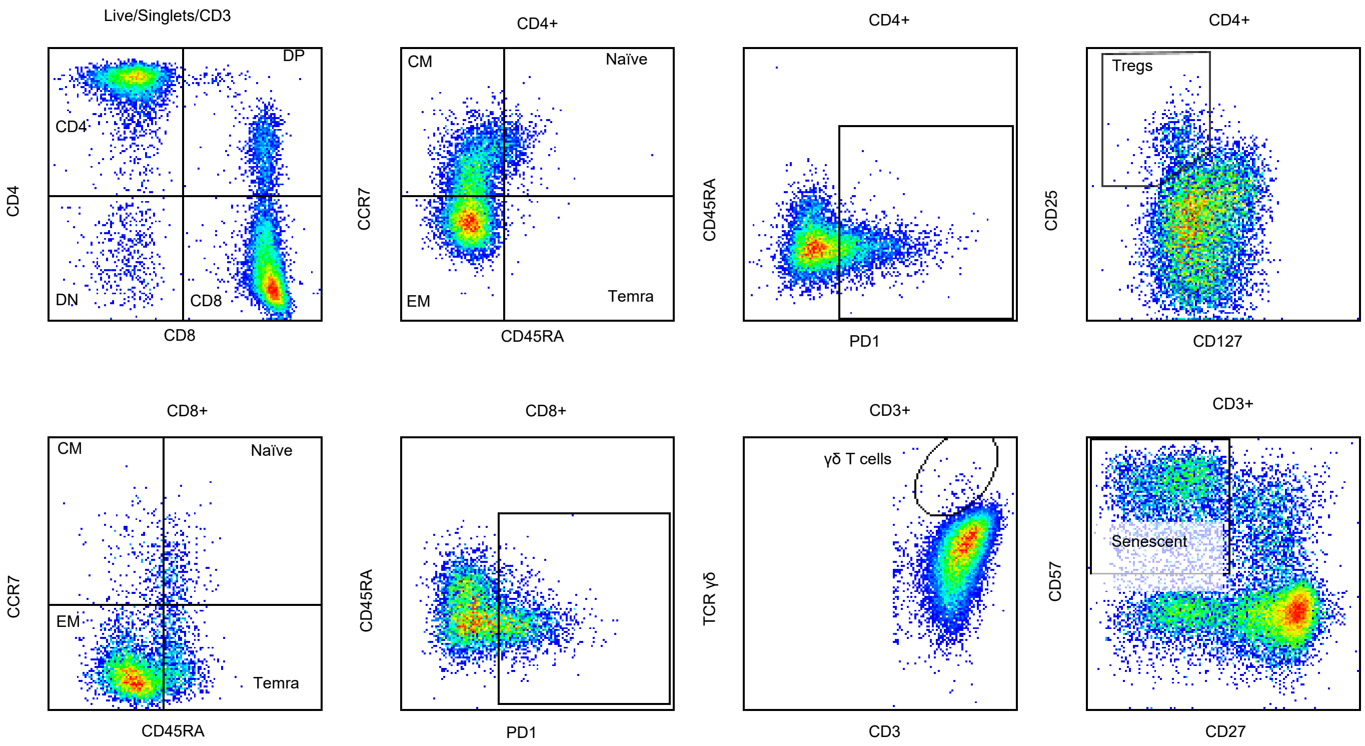

Supplementary Figure 4

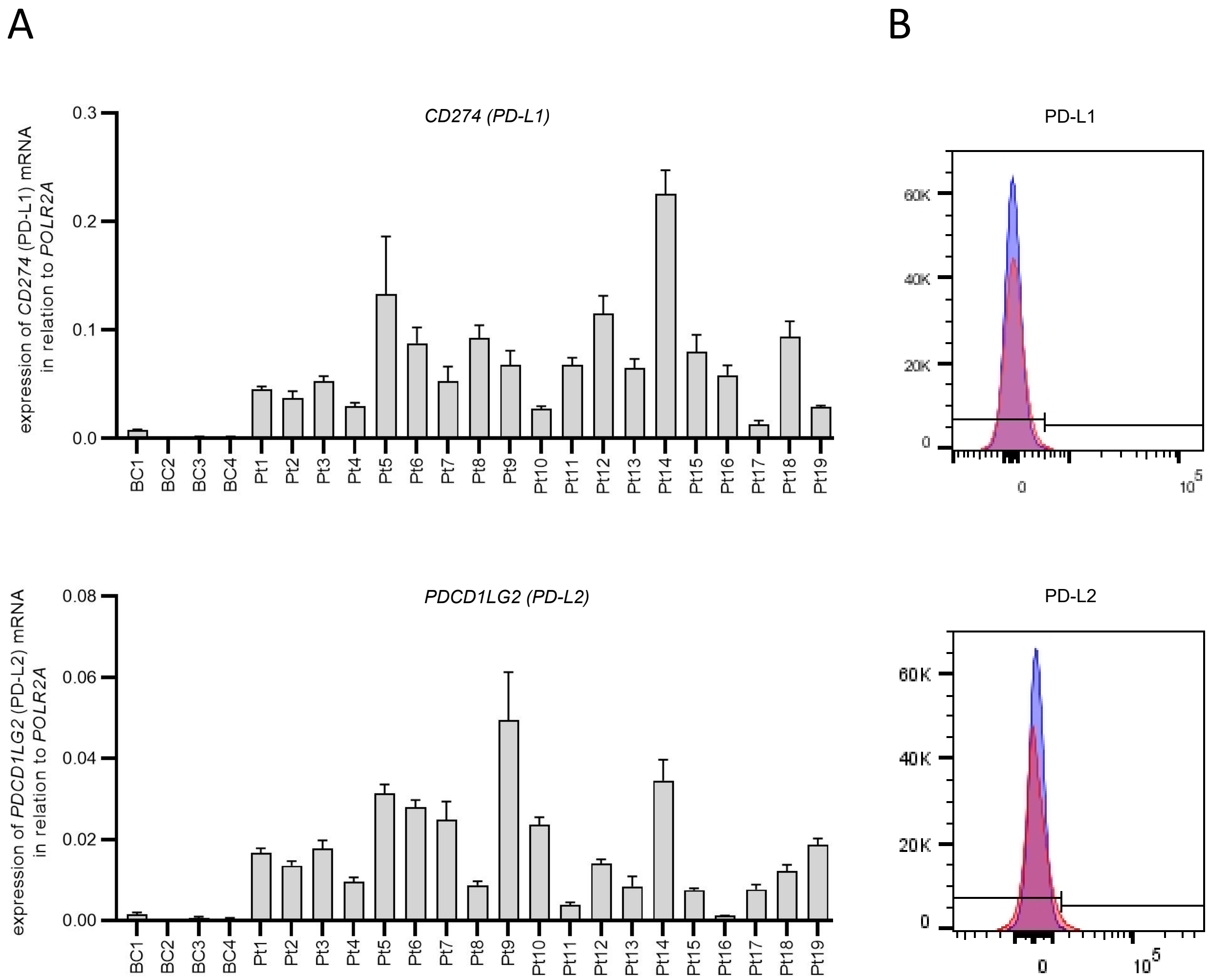

Supplementary Figure 5

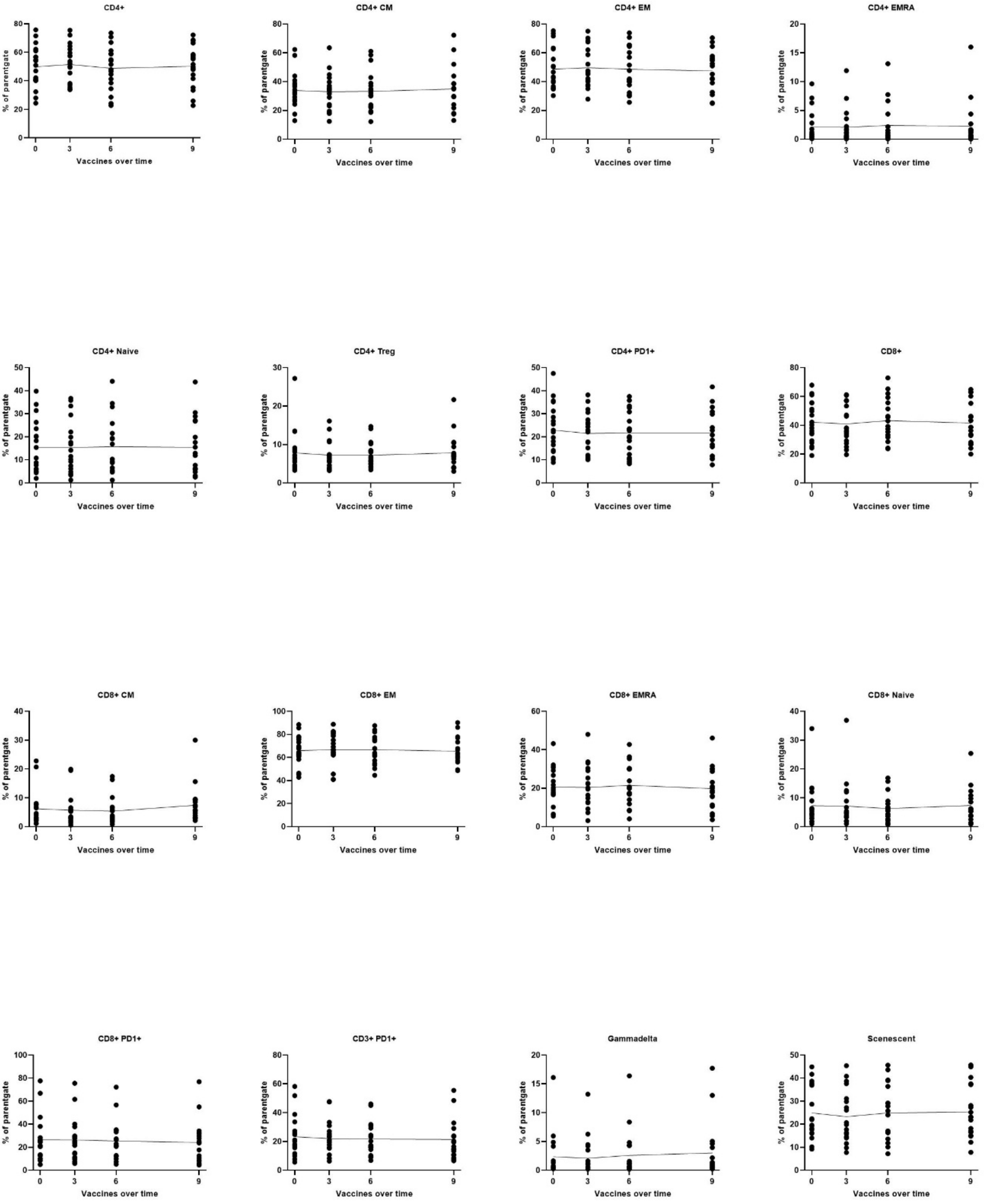

Supplementary Figure 6

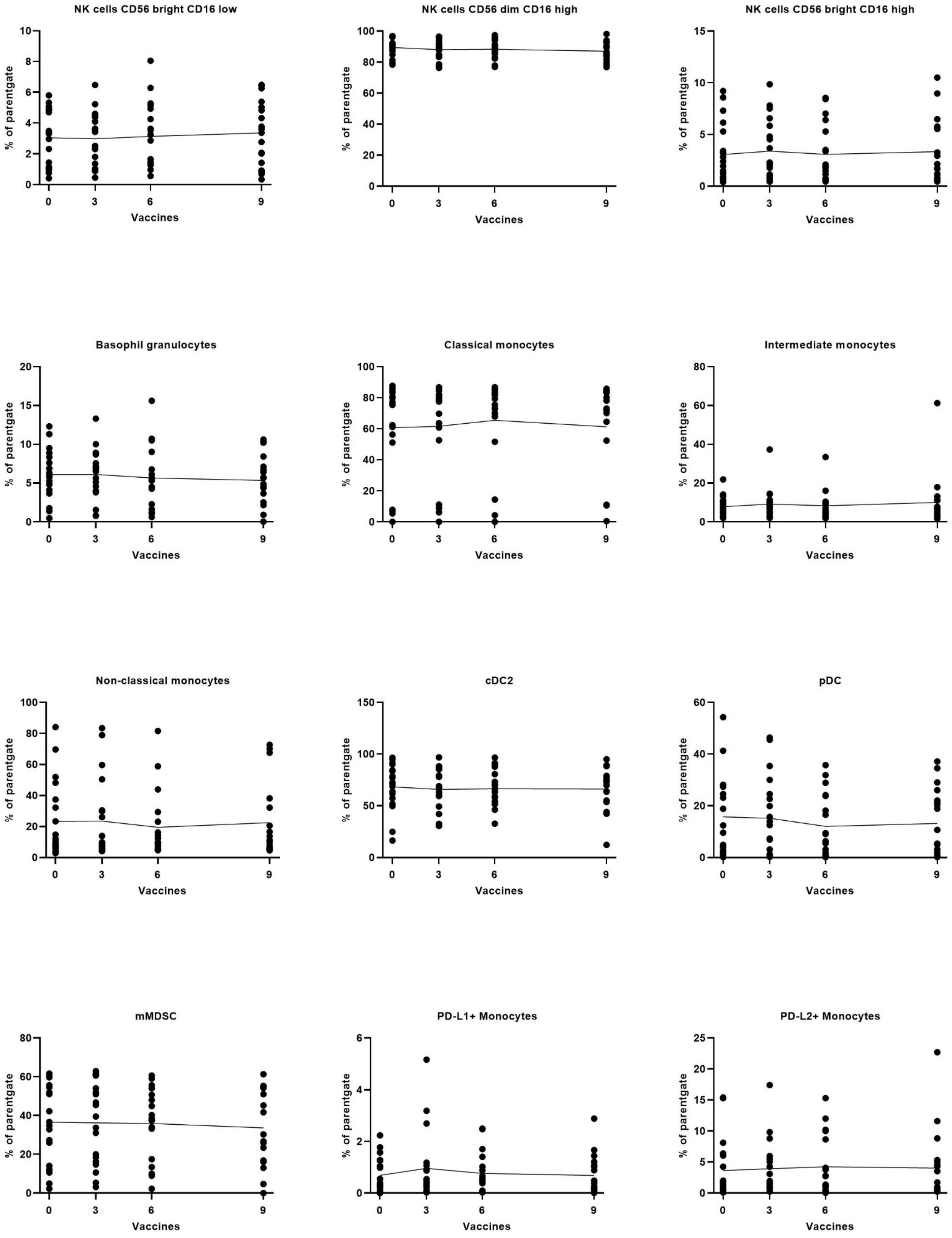

Supplement: Supplementary file 1 [file DataSheet_1.pdf]
