# Supplementary material for: Anti-PD-L1/PD-L2 therapeutic vaccination in untreated chronic lymphocytic leukemia patients with unmutated IgHV
Source: Front Oncol. 2022 Nov 22;12:1023015. doi: 10.3389/fonc.2022.1023015 (PMC9723164; doi:10.3389/fonc.2022.1023015)
Supplement: Supplementary file 2 [file DataSheet_2.pdf]

Supplementary material

Legends for supplementary figures and tables

Supplemental table 1: Inclusion and exclusion criteria

Supplemental figure 1: Treatment schedule

Supplemental table 2: List of antibodies

Supplemental figure 2: Gating strategy myeloid cells

Supplemental figure 3: Gating strategy T-cells

Supplemental figure 4: *PDCD1LG2* (PDL1) and *CD274 (*PDL2) expression by qPCR and flowcytometry

A: RT-qPCR analysis of *CD274* (PD-L1, top) and *PDCD1LG2* (PD-L2, bottom) expression. The relative gene expression of *CD274* or *PDCD1LG2* are normalized to the housekeeping gene *POLR2A* and given in arbitrary unit. Means of triplicates are shown with whiskers indicating standard deviations. See supplemental methods section

B: Flowcytometry analysis of the PD-L1 and PD-L2 surface expression (red) compared to the FMO control (blue).

Supplemental figure 5: Overview of immune populations during vaccination examined by flowcytometry – T-cells

Supplemental figure 6: Overview of immune populations during vaccination examined by flowcytometry – Myeloid cells

Supplemental methods

Total RNA extraction, cDNA synthesis and real time quantitative PCR (RT-qPCR)

RT-qPCR was used to determine the expression of *CD274* (PD-L1) and *PDCD1LG2* (PD-L2) and was performed at baseline for all patients. B-cells isolated from four healthy donors served as control. Healthy donor B-cells were isolated by MACS-sorting using the B-CLL isolation kit (Miltenyi Biotech). Total RNA isolation on PBMCs and B-cells was performed using the RNEasy Plus Mini Kit (Qiagen) according to manufacturer's instructions and eluded in 30µl RNAse-free water. The RNA concentration was estimated with NanoDrop2000 spectrophotometer. We used the High Capacity cDNA Reverse Transcription Kit (Applied Biosystems) to synthesize cDNA, using random primers and 1000ng RNA as input. The cDNA was diluted 1:1 prior to analysis and finally RT-qPCR analysis were performed using the TaqMan Gene Expression Assay on a Roche LightCycler 480 instrument. The assay was performed in technical triplicates for all primers and analyzed as previously described.(1) *POLR2A* served as the housekeeping gene, and all primers are listed in supplemental table 3. Ct values were set to 40 for low-expression samples with no amplification during the assay. qPCR was also used to assess minimal residual disease following published guidelines and validated by the EuroMRD consortium.(2,3) The clonal IGH sequence was used as target and standard curves were created from samples with a known CLL cell concentration of 10%, 1%, 0.1%, 0.05%, 0.01% and 0,005% diluted from a baseline sampled with an known fraction of CLL cells estimated by flowcytometry. The standard curves allowed us to estimate the percentage of CLL cells from the CT value. 500 ng per well of purified DNA from selected timepoints were run in triplicates. Albumin was used as control for DNA quality and quantity. The assay was performed on Quantstudio 7 (Thermo Fischer Scientific).Interpretation of the results also followed the EuroMRD guidelines.(3)

1. Bookout AL, Cummins CL, Mangelsdorf DJ. High-Throughput Real-Time Quantitative Reverse Transcription PCR. Curr Protoc Mol Biol. 2005; 71(1): 15.8.1-15.8.28.

2. Pott C, Brüggemann M, Ritgen M, van der Velden VHJ, van Dongen JJM, Kneba M. MRD detection in B-cell non-Hodgkin lymphomas using Ig gene rearrangements and chromosomal translocations as targets for real-time quantitative PCR. In: Küppers R, editor. Methods in Molecular Biology. (Humana Press, New York, NY); 2019. p. 199–228.

3. van der Velden VHJ, Cazzaniga G, Schrauder A, Hancock J, Bader P, Panzer-Grumayer ER, et al. Analysis of minimal residual disease by Ig/TCR gene rearrangements: Guidelines for interpretation of real-time quantitative PCR data. Leukemia. 2007; 21(4): 604–611.
